# Mutations in CHIP-associated genes at myeloid neoplasm diagnosis and risk of cardiovascular/cerebrovascular events

**DOI:** 10.1080/07853890.2026.2663092

**Published:** 2026-04-27

**Authors:** Yao Wang, Dewan Zhao, Yulong Chen, He Xu, Guirong Song, Xiuli Sun

**Affiliations:** ^a^ Department of Hematology, The First Affiliated Hospital of Dalian Medical University, Dalian, China; ^b^ School of Public Health, Dalian Medical University, Dalian, China

**Keywords:** Myeloid neoplasms, clonal hematopoiesis, somatic mutations, cardiovascular events, cerebrovascular events

## Abstract

**Background:**

Clonal hematopoiesis of indeterminate potential (CHIP)-associated somatic mutations are associated with increased cardiovascular and cerebrovascular risk in the general population; whether mutations detected at myeloid neoplasm (MN) diagnosis predict incident cardiovascular and cerebrovascular events (CCVEs) is unclear.

**Methods:**

We retrospectively studied 203 adults with newly diagnosed myelodysplastic neoplasms, myelodysplastic/myeloproliferative neoplasms, or acute myeloid leukemia unfit for intensive chemotherapy (2016–2023) who underwent pretreatment next-generation sequencing. Cause-specific hazard models adjusted for cardiovascular risk factors assessed associations between CHIP-associated gene mutations and incident CCVEs, including age-stratified and age-by-mutation interaction analyses.

**Results:**

Median age was 68 years (IQR, 61–75); 127 (62.6%) were male; 141 (69.5%) carried ≥1 CHIP-associated gene mutation, most commonly *ASXL1*, *TET2*, *TP53,* or *DNMT3A*. Over median 12-month follow-up, 74 (36.5%) experienced a first CCVE. Any CHIP-associated gene mutation was associated with CCVE risk in univariable analysis; this association was not significant after multivariable adjustment (adjusted hazard ratio [aHR], 1.58; 95% CI, 0.87–2.88; *p*=0.13). In joint age-mutation analyses, mutation-positive patients aged ≥70 years had the highest CCVE risk (aHR, 2.77; 95% CI, 1.17–6.56), whereas younger mutation-positive patients showed a nonsignificant trend (aHR, 2.16; 95% CI, 0.90–5.20; *p*=0.085); interaction was not significant (*p*=0.29). In multivariable models, CHIP-associated gene mutations, higher-risk MN, and incident CCVEs were each associated with higher all-cause mortality.

**Conclusions:**

CHIP-associated gene mutations at MN diagnosis were not independently associated with incident CCVEs. Older mutation-positive patients had higher observed CCVE risk, predominantly heart failure, with limited precision. These exploratory findings warrant prospective multicenter validation.

## Introduction

Clonal hematopoiesis (CH) is increasingly recognized as a shared risk factor for myeloid malignancies and cardiovascular disease (CVD) [1]. Clonal hematopoiesis of indeterminate potential (CHIP) refers to recurrent, malignancy-associated somatic mutations in individuals without morphologic evidence of hematologic neoplasms [2]. Because CHIP is defined in individuals without morphologic evidence of hematologic neoplasms, variants detected at MN diagnosis are not considered ‘CHIP’ in the clinical sense. Throughout this manuscript, we therefore use the term ‘mutations in CHIP-associated genes’ to refer to somatic mutations in canonical CH/CHIP driver genes detected at MN diagnosis, without implying a premalignant CHIP cohort or clonal origin outside the neoplasm.

CH is prevalent across populations. Large-scale sequencing studies of the UK Biobank cohort have demonstrated an age-dependent escalation in both variant allele frequency (VAF) and the prevalence of canonical CH driver mutations [3]. In individuals without hematologic malignancies, CH is associated with a 30–40% increase in all-cause mortality, primarily due to cardiovascular causes rather than progression to hematologic neoplasms [4,5]. Specifically, CH has been linked to a two-fold higher risk of coronary artery disease, a four-fold higher risk of premature myocardial infarction, and a 2.6-fold elevated risk of stroke [5–7]. Furthermore, CH has been associated with worse clinical outcomes in patients with chronic heart failure and an increased risk of arrhythmias [8,9].

Patients with myeloid neoplasms (MN) frequently develop cardiovascular and cerebrovascular events (CCVEs) during the course of their disease and treatment; however, whether mutations in CHIP-associated genes detected at MN diagnosis provide incremental prognostic information for incident CCVEs beyond conventional cardiovascular risk factors and prior cardiovascular/cerebrovascular disease remains uncertain, particularly in older, comorbidity-burdened populations who are unfit for intensive chemotherapy [10–13]. Therefore, we conducted a single-center retrospective cohort study of newly diagnosed MDS, MDS/MPN, and AML unfit for intensive therapy who underwent uniform pretreatment NGS, and we used competing-risk and cause-specific hazard modeling to assess associations between prespecified mutations in CHIP-associated genes and subsequent CCVEs as well as all-cause mortality, including age-stratified and age-by-mutation analyses.

## Methods

### Study population

This single-center retrospective cohort enrolled 203 adult patients (≥18 years) with newly diagnosed MN at the First Affiliated Hospital of Dalian Medical University between October 2016 and December 2023. The study population included patients with myelodysplastic neoplasms (MDS), myelodysplastic/myeloproliferative neoplasms (MDS/MPN), and acute myeloid leukemia (AML) unfit for intensive chemotherapy (hereafter, unfit AML), which was determined by a comprehensive assessment of age, performance status, and comorbidities. All patients underwent pretreatment next-generation sequencing (NGS) of bone marrow aspirate and/or peripheral blood. This study was approved by the Institutional Review Board of the First Affiliated Hospital of Dalian Medical University (Approval No: PJ-KS-KY-2024-413) and was conducted in accordance with the principles of the Declaration of Helsinki. Written informed consent was obtained at admission or at treatment initiation using the hospital’s general research consent, covering the use of de-identified clinical data for future research (including this study). For deceased participants, consent had been obtained during their lifetime.

Eligibility required pretreatment bone marrow and/or peripheral blood samples with qualified NGS and complete baseline and follow-up data. Patients with acute promyelocytic leukemia (APL) or incomplete records were excluded.

### Data collection

Demographic characteristics, baseline cardiovascular risk factors (CVRFs) (including diabetes mellitus, smoking, dyslipidemia, hypertension, and obesity), history of cardiovascular and cerebrovascular disease (CCVD) (including coronary artery disease, heart failure [HF], atrial fibrillation [AF], ischemic stroke, or peripheral artery disease [PAD]), and MN-related variables (including risk stratification and treatment strategies) were extracted from electronic records and supplemented by structured telephone interviews when needed; complete data were available for 203 patients. Follow-up was censored on September 30, 2024.

Patients were categorized into two age groups using 70 years as the cutoff value based on prior literature (patients aged ≥70 years were classified as the older group, while those <70 years comprised the younger group) [14–16]. At diagnosis, myeloid neoplasms (MN) were assigned to harmonized risk groups for analysis. Higher-risk MN comprised (i) higher-risk MDS, defined strictly by IPSS-R risk categories (intermediate, high, or very high), (ii) CMML with intermediate-2 or high risk by CPSS, and (iii) AML unfit for intensive chemotherapy, defined by treating physicians based on age, performance status, and comorbidity burden. Lower-risk MN included (i) lower-risk MDS, defined strictly by IPSS-R categories (very low or low), and (ii) MDS/MPN overlap neoplasms with a clinically indolent phenotype at diagnosis. MDS/MPN cases were considered clinically indolent if they lacked aggressive/proliferative features, defined as bone marrow blasts <5% and peripheral blood blasts <2%, WBC <13 × 10^9^/L, absence of symptomatic or progressive splenomegaly, and no requirement for cytoreductive therapy at presentation.

### Clinical outcomes

The primary outcome was a composite CCVE endpoint including (1) new-onset or worsening HF, (2) acute coronary syndrome (ACS), (3) coronary revascularization, (4) ischemic stroke, (5) venous thromboembolism, and (6) cardiovascular death. The secondary outcome was all-cause mortality. Detailed definitions of all outcomes are provided in the Supplemental Appendix.

### DNA analysis

NGS was performed on 184 bone marrow and 19 peripheral blood samples when marrow aspiration was inadequate. All samples were analyzed using an amplicon sequencing method targeting myeloid hotspot mutations. We focused on a prespecified subset of commonly reported CHIP-associated genes that were consistently covered across panels (DNMT3A, TET2, ASXL1, JAK2, TP53, SRSF2, SF3B1). Mutations were defined as pathogenic variants with variant allele frequencies (VAF) ≥2%. All panels covered these prespecified CHIP-associated genes. For patients harboring two or more mutations within a single gene, only the highest-VAF variant was retained for subsequent analysis.

### Statistical analysis

Baseline characteristics were summarized as medians with interquartile ranges (IQR) for non-normally distributed continuous variables and as frequencies with percentages for categorical variables. Between-group comparisons were performed using the Mann–Whitney U test for non-normally distributed variables and either the χ^2^ test or Fisher’s exact test for categorical variables, as appropriate.

Cause-specific hazard regression models adjusted for age group, sex, diabetes mellitus, dyslipidemia, hypertension, obesity, smoking status, prior CCVD, and MN risk group were used to evaluate associations between mutations in CHIP-associated genes and the risks of CCVEs. The analysis included: (1) a full-cohort assessment; (2) age-stratified analyses (<70 and ≥70 years); (3) an evaluation of the combined effects of age group and CHIP-associated gene mutation status; and (4) gene-specific analyses for individual mutations in CHIP-associated genes in the overall cohort.

Furthermore, to evaluate whether associations differed by age, an interaction term between age group and CHIP-associated gene mutation status was included in the cause-specific hazard models, and the interaction was evaluated using the Wald test. Cause-specific models were used for etiologic associations, whereas cumulative incidence functions (CIFs) described absolute risk under competing risks. CIFs for CCVEs were estimated using the Fine–Gray subdistribution hazard model, treating all-cause death without prior CCVE as a competing event, and between-group differences were assessed using Gray’s test. For all-cause mortality, CCVE status was modeled as a time-dependent covariate in Cox regression using the counting-process (start–stop) format. Hazard ratios (HRs) with 95% CIs are reported.

Statistical significance was defined as a two-sided *p*-value < 0.05. All statistical analyses were performed using R software (version 4.3.1).

## Results

### Patient enrollment and mutations in CHIP-associated genes

Among an initial cohort of 255 patients with newly diagnosed MN, 37 were excluded due to unavailable follow-up information, and 15 were excluded for incomplete baseline data. Consequently, a total of 203 newly diagnosed MN patients were included in the study, all of whom underwent NGS at the time of diagnosis and had complete baseline and follow-up data.

**Figure 1.**
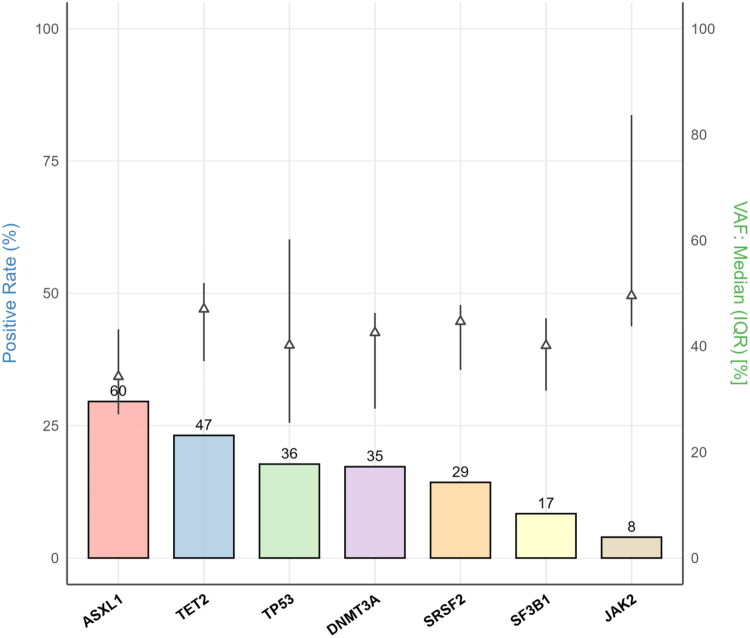
Distribution and VAF of mutations in CHIP-associated genes. Bars show the proportion of patients harboring each CHIP-associated gene mutation, and the numbers above bars indicate the absolute number of mutation carriers. Triangles with vertical lines depict the median and interquartile range of variant allele frequency (VAF) among carriers, referenced to the right y-axis. ASXL1, TET2, TP53, and DNMT3A were the most frequently mutated genes. CHIP = clonal hematopoiesis of indeterminate potential; IQR = interquartile range; VAF = variant allele frequency.

The median age of the cohort was 68 years (IQR 61–75), with 127 (62.6%) male patients. Genetic analysis identified a total of 232 mutations in CHIP-associated genes. Overall, 141 patients (69.5%) carried ≥1 mutation, and 66 patients (32.5%) carried ≥2. *ASXL1* was the most frequent (29.6%), followed by *TET2* (23.2%), *TP53* (17.7%), and *DNMT3A* (17.2%) (Figure 1). Lower mutation frequencies were observed for *SRSF2*, *SF3B1,* and *JAK2*.

### Baseline characteristics and outcomes by CHIP-associated gene mutation status

Patients’ baseline characteristics stratified by CHIP-associated gene mutation status (positive defined as ≥1 prespecified mutation) are summarized in Table 1. Patients with mutations in CHIP-associated genes were significantly older than those without such mutations (median age 70 years vs. 64 years, *p* < 0.001) and exhibited a higher proportion of males (68.8% vs. 48.4%, *p* = 0.006). The prevalence of prior CCVDs was comparable between the two groups (31.9% in the mutation-positive group vs. 29.0% in the mutation-negative group, *p* = 0.68). CVRFs were similar between groups, including obesity, dyslipidemia, diabetes mellitus, smoking, alcohol use, and hypertension. Mutations in CHIP-associated genes were significantly more common in patients with higher-risk MN (77.2%) compared to those with lower-risk (53.7%; *p* < 0.001).

**Table 1.** Characteristics of patients stratified by CHIP-associated mutation status.

	All (*n* = 203)	CHIP-associated mutation-negative (*n* = 62)	CHIP-associated mutation-positive (*n* = 141)	*P* value
Age, y	68.00 (61.00, 75.00)	62.00 (48.00, 71.00)	70.00 (65.00, 76.00)	<0.001
Age group				0.002
<70y	111 (54.68)	44 (70.97)	67 (47.52)	
≥70y	92 (45.32)	18 (29.03)	74 (52.48)	
Sex				0.006
Female	76 (37.44)	32 (51.61)	44 (31.21)	
Male	127 (62.56)	30 (48.39)	97 (68.79)	
BMI, kg/m^2^	23.81 (21.48, 26.30)	23.95 (22.04, 26.17)	23.67 (21.36, 26.42)	0.49
Overweight (BMI ≥ 24)	99 (48.77)	31 (50.00)	68 (48.23)	0.82
Obesity (BMI ≥ 28)	28 (13.79)	9 (14.52)	19 (13.48)	0.84
Diabetes mellitus	43 (21.18)	10 (16.13)	33 (23.40)	0.24
Hypertension	68 (33.50)	18 (29.03)	50 (35.46)	0.37
Dyslipidemia	157 (77.34)	45 (72.58)	112 (79.43)	0.28
Hemoglobin, g/L	71.00 (59.00, 94.00)	68.50 (54.00, 94.00)	74.00 (63.00, 94.00)	0.23
Smoking	66 (32.51)	15 (24.19)	51 (36.17)	0.093
Drinking	45 (22.17)	12 (19.35)	33 (23.40)	0.52
MN risk group				<0.001
Lower-risk MN	67 (33.00)	31 (50.00)	36 (25.53)	
Higher-risk MN	136 (67.00)	31 (50.00)	105 (74.47)	
Prior CCVD^a^	63 (31.03)	18 (29.03)	45 (31.91)	0.68
Prior CAD	33 (16.26)	10 (16.13)	23 (16.31)	0.97
Prior HF	24 (11.82)	9 (14.52)	15 (10.64)	0.43
Prior AF	6 (2.96)	3 (4.84)	3 (2.13)	0.37
Prior stroke	18 (8.87)	4 (6.45)	14 (9.93)	0.42
Prior PAD	1 (0.49)	1 (1.61)	0 (0.00)	0.30
CCVEs	74 (36.45)	15 (24.19)	59 (41.84)	0.016

Data are presented as n (%) or median (IQR). ^a^Prior CCVD includes coronary artery disease, heart failure, atrial fibrillation, ischemic stroke, or peripheral artery disease. AF = atrial fibrillation; BMI = body mass index; CAD = coronary artery disease; CCVD = cardiovascular and cerebrovascular disease; CCVE = cardiovascular and cerebrovascular event; CHIP = clonal hematopoiesis of indeterminate potential; HF = heart failure; MN = myeloid neoplasms; PAD = peripheral artery disease.

### Incidence of CCVEs during follow-up

During a median follow-up period of 12 months (IQR 6.0–25.0 months), 74 out of 203 patients (36.5%) experienced at least one CCVE. These events comprised 43 cases of new-onset or worsening HF, 17 cardiovascular deaths, 6 ischemic strokes, 4 cases of ACS, 1 coronary revascularization, and 3 venous thromboembolisms. Nine patients experienced a second event (including 4 ischemic strokes, 3 cardiovascular deaths, 1 worsening HF, and 1 venous thromboembolism). The CHIP-associated gene mutation–positive group exhibited a significantly higher incidence of CCVEs compared to the negative group (41.8% vs. 24.2%, *p* = 0.016) (Table 1). Baseline clinical characteristics stratified by the occurrence of CCVEs are summarized in Supplemental Table 1.

### Associations between mutations in CHIP-associated genes and CCVEs

In the univariable analysis, the presence of mutations in CHIP-associated genes was significantly associated with a 1.98-fold increased risk of CCVEs (HR 1.98, 95% CI 1.12–3.50). Similarly, advanced age (≥70 years) was a strong predictor, associated with a 1.88-fold increased risk (HR 1.88, 95% CI 1.20–2.96). Additionally, baseline dyslipidemia, history of diabetes mellitus, hypertension, prior CCVD, and higher-risk MN were also associated with increased CCVE risk (Table 2).

**Table 2.** Cause-specific hazard regression analysis on risks of CCVEs.

Variables	Univariable analysis		Multivariable analysis	
	HR (95% CI)	*P* Value	HR (95% CI)	*P* value
Age group, ≥70 y	1.88 (1.20–2.96)	0.006	1.44 (0.86–2.41)	0.16
Sex, male	1.21 (0.76–1.95)	0.42	0.77 (0.43–1.38)	0.38
Diabetes mellitus	1.73 (1.07–2.81)	0.026	1.40 (0.83–2.35)	0.21
Dyslipidemia	2.00 (1.10–3.66)	0.024	1.63 (0.89–2.97)	0.12
Hypertension	1.90 (1.21–2.99)	0.005	1.39 (0.83–2.32)	0.21
Obesity	1.47 (0.82–2.63)	0.20	1.28 (0.66–2.47)	0.46
Smoking	1.29 (0.81–2.04)	0.28	1.36 (0.74–2.51)	0.32
Prior CCVD	1.75 (1.11–2.74)	0.015	1.35 (0.77–2.34)	0.29
Higher-risk MN	1.66 (1.00–2.73)	0.049	1.44 (0.88–2.35)	0.15
CHIP-associated mutation	1.98 (1.12–3.50)	0.019	1.58 (0.87–2.88)	0.13

Values are cause-specific hazard ratios (HRs) with 95% confidence intervals. Abbreviations: CHIP = clonal hematopoiesis of indeterminate potential; CCVD = cardiovascular and cerebrovascular disease; CCVE = cardiovascular and cerebrovascular event; HR = hazard ratio; MN = myeloid neoplasms.

However, in the multivariable model adjusted for prespecified covariates, the association for mutations in CHIP-associated genes was no longer statistically significant (adjusted HR 1.58, 95% CI 0.87–2.88, *p* = 0.13), with wide confidence intervals. A notable parallel change was observed for the age variable, which also lost its independent statistical significance in the adjusted model (adjusted HR 1.44, 95% CI 0.86–2.41, *p* = 0.16). Similarly, a history of diabetes mellitus, dyslipidemia, hypertension, prior CCVD, and MN risk group also ceased to exhibit statistically significant associations in the multivariable analysis (Table 2).

Given the strong association between age and both mutation status and CCVE risk, we performed age-stratified and joint age-mutation analyses to assess the consistency of associations across age groups. Among the 111 patients aged <70 years, the presence of mutations in CHIP-associated genes tended to be associated with increased CCVE risk (HR 2.22, 95% CI 0.90–5.48, *p* = 0.084). No statistically significant association was observed in patients aged ≥70 years (HR 1.17, 95% CI 0.55–2.47, *p* = 0.69) (Table 3). However, this interaction between age group and CHIP-associated gene mutation status was not statistically significant in the multivariable model (*p* for interaction = 0.29). Therefore, while the HR point estimates suggest a potential stronger effect of CHIP-associated gene mutation status in younger patients, the study lacks sufficient evidence to conclusively state that the effect differs by age. Additionally, higher-risk MN also tended to be associated with increased CCVE risk in patients aged <70 years (HR 2.17, 95% CI 0.97–4.87, *p* = 0.059).

**Table 3.** Multivariable cause-specific hazard models for CCVE risk by age group and CHIP status.

	Younger patients (<70 years)		Older patients (≥70 years)	
	HR (95% CI)	*P* Value	HR (95% CI)	*P* value
Sex, male	0.82 (0.36–1.87)	0.64	0.85 (0.37–1.96)	0.70
Diabetes mellitus	1.15 (0.45–2.96)	0.77	1.54 (0.81–2.93)	0.19
Dyslipidemia	1.20 (0.51–2.83)	0.67	2.43 (0.95–6.17)	0.062
Hypertension	1.49 (0.68–3.26)	0.32	1.46 (0.76–2.79)	0.26
Obesity	1.19 (0.44–3.22)	0.73	1.14 (0.44–2.91)	0.79
Smoking	0.99 (0.37–2.67)	0.98	1.50 (0.69–3.30)	0.31
Prior CCVD	1.83 (0.74–4.52)	0.19	1.15 (0.58–2.28)	0.68
Higher-risk MN	2.17 (0.97–4.87)	0.059	0.98 (0.50–1.93)	0.96
CHIP-associated mutations	2.22 (0.90–5.48)	0.084	1.17 (0.55–2.47)	0.69

Values are adjusted cause-specific hazard ratios (HRs) with 95% confidence intervals for CCVEs. Abbreviations: CCVD = cardiovascular and cerebrovascular disease; CCVE = cardiovascular and cerebrovascular event; CHIP = clonal hematopoiesis of indeterminate potential; CI = confidence interval; HR = hazard ratio; MN = myeloid neoplasms.

**Figure 2.**
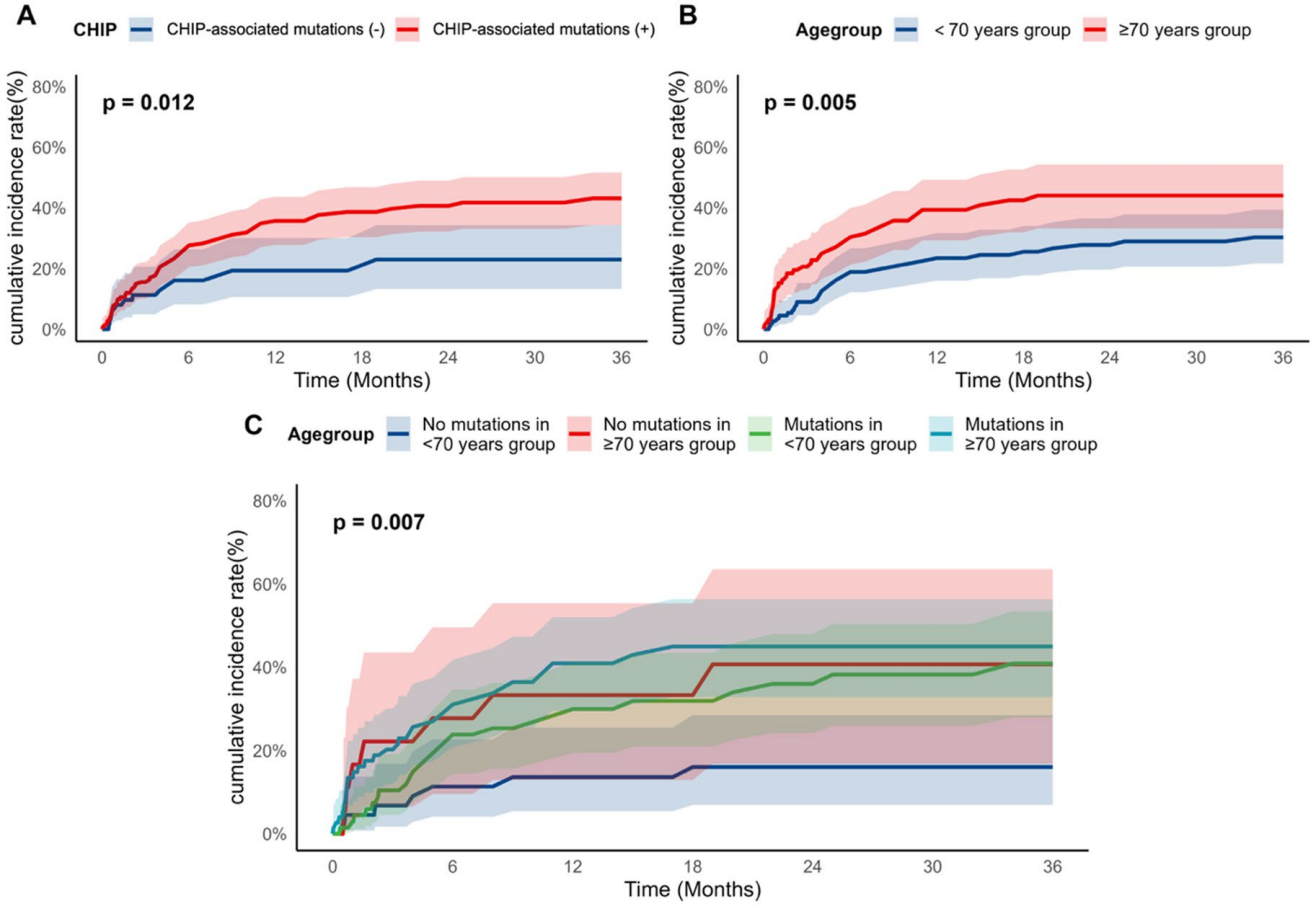
Cumulative incidence curves of CCVEs. This figure shows cumulative incidence curves of cardiovascular and cerebrovascular events (CCVEs) estimated with the Fine–Gray subdistribution hazard model, accounting for death without prior CCVE as a competing event. Panel A compares patients with vs. without mutations in CHIP-associated genes. Panel B compares age groups (<70 vs. ≥70 years). Panel C shows curves stratified jointly by age group and CHIP-associated gene mutation status. Shaded areas indicate 95% confidence intervals. CCVE = cardiovascular and cerebrovascular event; CHIP = clonal hematopoiesis of indeterminate potential.

Further, patients were cross-classified into four groups based on their age (<70 or ≥70 years) and CHIP-associated gene mutation status (positive or negative), with younger patients without mutations serving as the reference group. In univariable analyses, all groups showed a significantly elevated risk of CCVEs compared to the reference group (Table 4, Figure 2). After multivariable adjustment, the highest risk was observed in older patients with mutations in CHIP-associated genes, which remained statistically significant (adjusted HR 2.77, 95% CI 1.17–6.56). Younger patients with mutations showed a strong, borderline trend toward an increased risk (adjusted HR 2.16, 95% CI 0.90–5.20, *p* = 0.085). In contrast, older patients without mutations no longer had a significantly different risk from the reference group (adjusted HR 2.37, 95% CI 0.82–6.86). In the joint age-mutation status cross-classification, older mutation-positive patients had the highest adjusted hazard; however, confidence intervals were wide and findings should be interpreted as exploratory.

**Table 4.** Combined effects of age group and CHIP-associated mutations on CCVEs.

Age-CHIP groups	Univariable analyses		Multivariable analyses^a^	
	HR (95% CI)	*P* value	HR (95% CI)	*P* value
Younger age group without CHIP	REF	–	REF	–
Older age group without CHIP	3.56 (1.28–9.86)	0.015	2.37 (0.82–6.86)	0.11
Younger age group with CHIP	2.69 (1.16–6.26)	0.022	2.16 (0.90–5.20)	0.085
Older age group with CHIP	3.74 (1.64–8.55)	0.002	2.77 (1.17–6.56)	0.021

^a^ The Cause-specific hazard regression model was adjusted for covariates, including sex, diabetes mellitus, dyslipidemia, hypertension, obesity, smoking, history of cardiovascular disease, and MN risk groups. Abbreviations: CCVE = cardiovascular and cerebrovascular event; CHIP = clonal hematopoiesis of indeterminate potential; CI = confidence interval; HR = hazard ratio.

### Specific mutations in CHIP-associated genes and CCVEs

In multivariable cause-specific hazard models, DNMT3A (HR 1.87, 95% CI 0.98–3.59; *p* = 0.058) and TET2 (HR 1.87, 95% CI 0.99–3.52; *p* = 0.052) showed borderline, exploratory associations with CCVE risk. Because analyses were uncorrected for multiple testing and based on limited event counts, these findings should be considered hypothesis-generating (Table 5).

**Table 5.** Multivariable cause-specific hazard models for single CHIP-associated mutations and CCVE risk.

	HR (95% CI)	*P* value
Age group, ≥70 y	1.55 (0.91–2.61)	0.10
Sex, male	0.78 (0.44–1.41)	0.42
Diabetes mellitus	1.48 (0.89–2.46)	0.13
Dyslipidemia	1.79 (0.95–3.38)	0.070
Hypertension	1.22 (0.70–2.11)	0.48
Obesity	1.50 (0.80–2.84)	0.21
Smoking	1.60 (0.84–3.06)	0.15
Prior CCVD	1.62 (0.90–2.92)	0.11
Higher-risk MN	1.54 (0.92–2.57)	0.10
CHIP-associated mutations		
*DNMT3A*	1.87 (0.98–3.59)	0.058
*TET2*	1.87 (0.99–3.52)	0.052
*ASXL1*	0.70 (0.38–1.29)	0.26
*TP53*	1.61 (0.83–3.12)	0.16
*JAK2*	0.88 (0.23–3.45)	0.86
*SF3B1*	1.48 (0.66–3.31)	0.34
*SRSF2*	0.71 (0.33–1.52)	0.38

Abbreviations: CHIP = clonal hematopoiesis of indeterminate potential; CI = confidence interval; CCVD = cardiovascular and cerebrovascular disease; CCVE = cardiovascular and cerebrovascular event; HR = hazard ratio; MN = myeloid neoplasms.

### Mutations in CHIP-associated genes and all-cause mortality

During follow-up, 136 (67.0%) patients died, with a median survival of 9.00 months (IQR 3.64–15.50). Among those who died, the median age at diagnosis was 70 years (IQR 63.0–75.0), 29 (21.3%) were mutation-negative, 57 (41.9%) carried one mutation, and 50 (36.8%) carried ≥2. Most deaths occurred in higher-risk MN (108/136), compared with 28/136 in lower-risk MN. In the time-to-death analysis, time-updated CCVE status was associated with a higher risk of all-cause mortality (HR 2.79, 95% CI 1.80–4.34; *p* < 0.001; Table 6).

**Table 6.** Determinants of all-cause mortality: Multivariable cox proportional hazards analysis.

	HR (95% CI)	*P* Value
CCVEs^#^	2.79 (1.80–4.34)	<0.001
Age group, ≥70y	1.20 (0.84–1.73)	0.320
Sex, male	1.17 (0.75–1.84)	0.487
Diabetes mellitus	0.86 (0.54–1.38)	0.540
Dyslipidemia	1.44 (0.93–2.23)	0.100
Hypertension	0.70 (0.46–1.04)	0.078
Obesity	1.02 (0.60–1.73)	0.943
Smoking	0.84 (0.54–1.32)	0.456
Prior CCVD	1.42 (0.95–2.12)	0.089
Higher-risk MN	2.54 (1.62–3.98)	<0.001
CHIP-associated mutations	1.78 (1.13–2.79)	0.013

^#^ CCVE was treated as a time-dependent covariate using the counting process approach. Abbreviations: CHIP = clonal hematopoiesis of indeterminate potential; CI = confidence interval; CCVD = cardiovascular and cerebrovascular disease; CCVEs = cardiovascular and cerebrovascular events; HR = hazard ratio; MN = myeloid neoplasms.

## Discussion

In newly diagnosed myeloid neoplasms, the presence of mutations in CHIP-associated genes showed an increased CCVE risk in univariable analyses, but the association was not statistically significant after multivariable adjustment. Joint age-mutation stratification suggested a higher observed risk among older mutation-positive patients, consistent with a hypothesis-generating pattern of higher observed risk when older age and mutation-positive status co-occur. Overall, these findings warrant external validation before clinical implementation.

After multivariable adjustment, the association was not statistically significant, and confidence intervals were wide, indicating limited precision. Given the modest sample size, we cannot distinguish true confounding from limited power or model uncertainty. Accordingly, the multivariable non-significance should be interpreted as inconclusive rather than evidence of no association. Joint age-mutation stratification provides a hypothesis-generating signal that warrants prospective validation.

These findings extend prior evidence that mutations in CHIP-associated genes confer cardiovascular vulnerability in the general population and in selected cancer cohorts. Large population studies have linked mutations in CHIP-associated genes to atherosclerotic cardiovascular disease, early-onset myocardial infarction, stroke, and heart failure, even after accounting for traditional risk factors [6,17]. In non-APL AML, Calvillo-Argüelles et al. reported a higher 3-year cumulative incidence of cardiovascular events in mutation-positive versus mutation-negative patients (22.3% vs. 15.7% overall), with a clearer separation among those receiving intensive chemotherapy but not in the non-intensive group [11]. In our broader MN cohort enriched for older patients and predominantly non-intensive therapy, absolute CCVE risk was higher and the prevalence of mutations in CHIP-associated genes was greater; the overall multivariable association of mutations in CHIP-associated genes with CCVEs was not statistically significant after adjustment, while joint age-mutation analyses suggested the highest hazards among older mutation-positive patients. These cross-cohort differences likely reflect heterogeneity in age and disease spectrum, treatment intensity, baseline comorbidity structure, and inflammatory exposure associated with mutations in CHIP-associated genes in chronic MN.

In AML, Kang et al. adjudicated symptomatic HF and found DNMT3A mutations associated with higher HF risk (1-year cumulative incidence 11.4% vs. 3.9%), with persistence after adjustment for age and anthracycline exposure and amplification by baseline cardiovascular risk factors; variant allele fraction was not associated with HF [18]. In our MN cohort, HF was the most common first CCVE presentation, and DNMT3A showed a borderline association with incident CCVEs after multivariable adjustment (adjusted HR 1.87; *p* = 0.058). Although endpoints (adjudicated HF vs. composite CCVEs) and treatment intensity differ, the phenotypic concordance is consistent with prior reports and may be biologically plausible; however, our gene-level signal remains exploratory given limited events and lack of multiple-testing correction. Complementing this, Naqvi et al. examined untreated MDS and reported gene-specific patterns of baseline cardiovascular comorbidity (e.g. DNMT3A with prior myocardial infarction; JAK2 with veno-occlusive disease) and highlighted TP53 and comorbidity burden as key determinants of overall survival, whereas TET2 was not associated with cardiovascular comorbidity in that cross-sectional framework [19]. These observations complement our study by contrasting baseline comorbidity profiling at diagnosis with our longitudinal assessment of incident CCVEs, and by underscoring that heterogeneity across studies likely reflects differences in design/endpoints, population and treatment mix, and covariate adjustment.

Mechanistic studies support the plausibility of gene-specific cardiovascular vulnerability conferred by mutations in CHIP-associated genes. TET2-mutant clonal hematopoiesis amplifies macrophage inflammasome signaling (NLRP3-IL-1β), promoting atherogenesis and adverse cardiac remodeling, whereas DNMT3A-mutant immune cells adopt pro-inflammatory programs that activate cardiac fibroblasts and foster interstitial fibrosis and remodeling [20–23]. TP53-mutant clonal hematopoiesis (TP53-CH) has been linked to systemic atherosclerosis through macrophage proliferative programs, and JAK2-CH has been consistently associated with arterial/venous thrombosis and endothelial dysfunction, highlighting potentially distinct cardiovascular phenotypes across CHIP-associated gene mutations [24–26]. Given limited event counts and heterogeneity of the literature, gene-level associations in our cohort should be interpreted as exploratory and require prospective validation.

Age may further modify the cardiovascular vulnerability associated with CHIP-associated gene mutation status. Prior studies link mutations in CHIP-associated genes to both atherosclerotic events and heart-failure phenotypes, including an association between TET2-mutant clonal hematopoiesis and HFpEF, which becomes more prevalent with advancing age [6,17]. In our cohort, CCVEs were predominantly heart-failure presentations and ischemic components were infrequent, limiting robust component-specific inference; serial assessment may be informative in selected high-risk oncology populations as clone size can evolve over time [27]. Accordingly, the primary CCVE composite was intended to reflect overall cardiovascular/cerebrovascular burden in this cohort rather than a single atherothrombotic pathway. Conventional major adverse cardiovascular events (MACE) (cardiovascular death, myocardial infarction (MI) or ACS, and ischemic stroke, with or without coronary revascularization) typically excludes heart-failure hospitalization and would not capture the dominant cardiovascular phenotype observed in our setting.

Our findings generate a testable hypothesis that CHIP-associated gene mutation status, together with age, may inform cardio-oncology risk assessment at MN diagnosis. However, given limited precision in this single-center retrospective cohort, any mutations in CHIP-associated genes-informed approach should be considered preliminary and requires prospective multicenter validation before clinical implementation. Pending validation, heightened awareness and selective cardiovascular evaluation (e.g. biomarkers and echocardiography when clinically indicated) may be reasonable in higher-risk patients, while general cardiovascular prevention should follow established guidelines [28]. Inflammatory biology associated with mutations in CHIP-associated genes also supports future trials of targeted preventive strategies (e.g. IL-1β-pathway modulation), but such approaches must be carefully evaluated in MN populations given cytopenias, infection risk, and potential drug-drug interactions [29,30].

Strengths of this study include uniform pretreatment NGS and a prespecified, clinically relevant composite CCVE endpoint with heart-failure adjudication. Key limitations include the retrospective single-center design with potential ascertainment bias (e.g. inpatient heart-failure events may be captured more completely than outpatient ischemic events). Although we anchored our harmonized risk grouping to validated disease-specific tools and objective clinical criteria, residual heterogeneity across MN entities (particularly the CMML spectrum and AML-related physiologic stress) may remain and limits subtype-specific inference. Residual confounding by treatment exposure (including time-varying transfusion burden, infections, and hospitalization intensity) is a major limitation; thus, findings should not be interpreted causally. In addition, the low frequency of non-heart-failure CCVE components limited event-type-specific analyses, modest power constrained gene-level analyses, and we relied on a single time-point CHIP-associated gene mutation status assessment without clonal dynamics or aggregate mutational burden. Although a broader gene panel was sequenced in routine care, our primary analyses were restricted a priori to these seven commonly reported CH/CHIP driver genes; therefore, findings may not generalize to less frequently reported CH-associated genes outside this prespecified set. We did not adjust for multiple comparisons across genes; therefore, gene-specific findings should be considered exploratory. These limitations constrain precision and generalizability and underscore the need for prospective multicenter validation.

## Conclusions

In newly diagnosed myeloid neoplasms, older patients with mutations in CHIP-associated genes showed the highest observed risk of incident CCVEs, dominated by heart failure presentations. Although the independent association of CHIP-associated gene mutation status with CCVEs was not statistically significant after multivariable adjustment, presence of mutations in CHIP-associated genes was associated with worse overall survival. These findings are hypothesis-generating and warrant prospective, multicenter validation.

## Supplementary Material

Supplemental Appendix.docx

## Data Availability

The datasets generated and/or analyzed during the current study are not publicly available due to institutional policies and patient privacy considerations but are available from the corresponding author on reasonable request.
