# Supplementary material for: Mutations in CHIP-associated genes at myeloid neoplasm diagnosis and risk of cardiovascular/cerebrovascular events
Source: Ann Med. 2026 Apr 27;58(1):2663092. doi: 10.1080/07853890.2026.2663092 (PMC13123072; doi:10.1080/07853890.2026.2663092)
Supplement: Supplemental Appendix.docx [file IANN_A_2663092_SM8347.docx]

**Supplemental Appendix**

**Title of the Manuscript:**

***Mutations in CHIP-associated Genes at Myeloid Neoplasm Diagnosis and Risk of Cardiovascular/Cerebrovascular Events***

**Contents of the Supplemental Appendix**

**Supplemental Methods**

**• Supplemental Methods 1. Outcome Definitions…………………………………………...2**

**Supplemental Tables**

**• Supplemental Table 1. Baseline Characteristics of Patients Stratified by the Occurrence of CCVEs……………………………………………………………………………………….3**

**Supplemental Methods 1. Outcome Definitions:**

**New-onset or worsening heart failure:**

1. New-onset heart failure: First occurrence of typical symptoms and signs of heart failure, and meeting at least one of the following objective criteria: (i) evidence of cardiac structural or functional abnormality; (ii) elevated natriuretic peptide levels; or (iii) invasive hemodynamics demonstrating a pulmonary capillary wedge pressure (PCWP) >15 mm Hg.

2. Worsening heart failure: Onset or progression of heart failure signs and symptoms in patients who are receiving treatment and were previously stable, requiring hospitalization, emergency department care, or the administration of intravenous diuretics in an outpatient clinic.

**Acute coronary syndrome:** Myocardial infarction or unstable angina, with either (i) coronary artery disease demonstrated on coronary angiography (≥70% stenosis in a major epicardial artery), or (ii) evidence of ischemia on noninvasive testing.

**Percutaneous or surgical coronary artery revascularization:** Any procedure performed to restore coronary blood flow in patients with significant epicardial coronary artery disease, via either percutaneous coronary intervention (PCI) or coronary artery bypass grafting (CABG).

**Ischemic stroke:** An acute episode of neurological dysfunction attributed to focal cerebral, spinal, or retinal infarction, confirmed by pathological, imaging, or other objective evidence of ischemic injury in a defined vascular distribution; or clinical evidence of focal ischemic injury affecting the brain, spinal cord, or retina, with symptoms lasting ≥24 hours or until death, after exclusion of alternative etiologies.

**Venous thromboembolism:** Deep vein thrombosis and/or pulmonary embolism confirmed by appropriate imaging, such as Doppler ultrasound, computed tomography pulmonary angiography, or ventilation/perfusion scanning.

**Cardiovascular death:** Death resulting from heart failure, myocardial infarction, or ischemic stroke.

**Supplemental Table 1. Baseline Characteristics of Patients Stratified by the Occurrence of CCVEs**

|  | **All**  **(n=203)** | **Absence of CCVEs**  **(n=129)** | **Occurrence of CCVEs**  **(n=74)** | ***P* Value** |
| --- | --- | --- | --- | --- |
| Age, y | 68.00 (61.00, 75.00) | 65.00 (56.00, 73.00) | 71.00 (65.00, 77.00) | <0.001 |
| Age group |  |  |  | 0.013 |
| <70y | 111 (54.68) | 79 (61.24) | 32 (43.24) |  |
| ≥70y | 92 (45.32) | 50 (38.76) | 42 (56.76) |  |
| Sex |  |  |  | 0.42 |
| Female | 76 (37.44) | 51.00 (39.53) | 25 (33.78) |  |
| Male | 127 (62.56) | 78 (60.47) | 49 (66.22) |  |
| BMI, kg/m^2^ | 23.81 (21.48, 26.30) | 24.05 (21.38, 26.17) | 23.43 (21.72, 26.75) | 0.85 |
| Overweight  (BMI≥24) | 99 (48.77) | 67 (51.94) | 32 (43.24) | 0.23 |
| Obesity (BMI≥28) | 28 (13.79) | 15 (11.63) | 13 (17.57) | 0.24 |
| Diabetes mellitus | 43 (21.18) | 21 (16.28) | 22 (29.73) | 0.024 |
| Hypertension | 68 (33.50) | 35 (27.13) | 33 (44.59) | 0.011 |
| Dyslipidemia | 157 (77.34) | 94 (72.87) | 63 (85.14) | 0.044 |
| Hemoglobin, g/L | 71.00 (59.00, 94.00) | 75.00 (59.00, 95.00) | 71.00 (62.00, 83.00) | 0.24 |
| Smoking | 66 (32.51) | 38 (29.46) | 28 (37.84) | 0.22 |
| Drinking | 45 (22.17) | 28 (21.71) | 17 (22.97) | 0.83 |
| MN subtype |  |  |  | 0.093 |
| Lower-risk MN | 67 (33.00) | 48 (37.21) | 19 (25.68) |  |
| Higher-risk MN | 136 (67.00) | 81 (62.79) | 55 (74.32) |  |
| Prior CCVD | 63 (31.03) | 32 (24.81) | 31 (41.89) | 0.011 |
| Prior CAD | 33 (16.26) | 14 (10.85) | 19 (25.68) | 0.006 |
| Prior HF | 24 (11.82) | 8 (6.20) | 16 (21.62) | 0.001 |
| Prior AF | 6 (2.96) | 3 (2.33) | 3 (4.05) | 0.67 |
| Prior stroke | 18 (8.87) | 9 (6.98) | 9 (12.16) | 0.21 |
| Prior PAD | 1 (0.49) | 1 (0.78) | 0 (0.00) | >0.999 |
| CHIP-associated  mutation (+) | 141 (69.46) | 82 (63.57) | 59 (79.73) | 0.016 |
| *DNMT3A* | 35 (17.24) | 18 (13.95) | 17 (22.97) | 0.10 |
| *TET2* | 47 (23.15) | 24 (18.60) | 23 (31.08) | 0.043 |
| *ASXL1* | 60 (29.56) | 38 (29.46) | 22 (29.73) | 0.97 |
| *JAK2* | 8 (3.94) | 5 (3.88) | 3 (4.05) | >0.999 |
| *TP53* | 36 (17.73) | 21 (16.28) | 15 (20.27) | 0.47 |
| *SRSF2* | 29 (14.29) | 17 (13.18) | 12 (16.22) | 0.55 |
| *SF3B1* | 17 (8.37) | 9 (6.98) | 8 (10.81) | 0.34 |

Abbreviations: AF = atrial fibrillation; BMI = body mass index; AML = acute myeloid leukemia; CAD = coronary artery disease; CCVD = cardiovascular and cerebrovascular diseases; CCVE = cardiovascular and cerebrovascular events; CHIP = Clonal hematopoiesis of indeterminate potential; CMML = chronic myelomonocytic leukemia; MDS = myelodysplastic neoplasms; MDS/MPN = myelodysplastic/myeloproliferative neoplasms; PAD = peripheral artery disease.
